# Social status and novelty drove the spread of online information during the early stages of COVID-19

**DOI:** 10.1038/s41598-021-99060-y

**Published:** 2021-10-11

**Authors:** Antonis Photiou, Christos Nicolaides, Paramveer S. Dhillon

**Affiliations:** 1 School of Economics and Management, University of Cyprus, 2109 Aglantzia, Nicosia Cyprus; 2 School of Information, University of Michigan, Ann Arbor, MI 48109 USA; 3 Initiative on the Digital Economy, Massachusetts Institute of Technology, Cambridge, MA 02142 USA

**Keywords:** Computational science, Computer science, Information technology

## Abstract

Access to online information has been crucial throughout the COVID-19 pandemic. We analyzed more than eight million randomly selected Twitter posts from the first wave of the pandemic to study the role of the author’s social status (Health Expert or Influencer) and the informational novelty of the tweet in the diffusion of several key types of information. Our results show that health-related information and political discourse propagated faster than personal narratives, economy-related or travel-related news. Content novelty further accelerated the spread of these discussion themes. People trusted health experts on health-related knowledge, especially when it was novel, while influencers were more effective at propagating political discourse. Finally, we observed a U-shaped relationship between the informational novelty and the number of retweets. Tweets with average novelty spread the least. Tweets with high novelty propagated the most, primarily when they discussed political, health, or personal information, perhaps owing to the immediacy to mobilize this information. On the other hand, economic and travel-related information spread most when it was less novel, and people resisted sharing such information before it was duly verified.

## Introduction

The COVID-19 pandemic has viciously engulfed the entire world since January 2020, representing a crisis of an order unseen in the recent past. While the world has been scrambling to come to order and restore normalcy, social media has served as an essential conduit of information exchange during this time while also creating channels of coordination between geographically distant individuals^1,2^. This is not surprising since, globally, two-thirds of Internet users use social media and a significant proportion of them use it as a source of health and science news^3^. The stay-at-home regulations, travel bans, and mask mandates enforced as part of COVID-19 globally created much chaos and uncertainty. And at the same time, the portending economic and travel impact of the pandemic induced anxiety. Twitter and other social media were the ultimate refuges of many people to access timely and accurate information and share their personal experiences^4^.

Early studies investigating COVID-19 information sharing on Twitter show a high correlation between tweet volume and the number of cases reported^3^, and that the announcement of the first COVID-19 case led to an increase in information-seeking regarding the virus but not about the treatment or non-pharmaceutical interventions^4^. Studies of Reddit posts revealed people to be feeling more socially connected to their family during the pandemic outbreak than before the pandemic^5,6^. There was also evidence regarding the evolution of emotions from fear to anger during the initial stages of the pandemic, with the anger being related to xenophobia. However, the discourse shifted to the stay-at-home practices as the pandemic progressed^7^.

An uptick in the spread of misinformation was also observed by several studies that analyzed Twitter conversations^3,8^. The prevalence of misinformation was higher among the unverified accounts than among verified or healthcare-related accounts^9,10^. The connection between the number of likes or retweets and the veracity of the COVID-19 related content, however, remains ambiguous^9–11^. Interestingly, it turns out that most of the misinformation regarding COVID-19 circulating on Twitter involved misinterpretation or misleading rephrasing of the true information rather than being completely fabricated^11^.

These studies collectively describe the social, psychological, behavioral, and mental health impacts of the pandemic on people worldwide, as revealed by their social media activity. Though these equilibrium social interactions reveal subtle patterns at the micro and macro-level, they fail to capture the turbulence of information flow in the early stages of the pandemic when the world was still grappling with a “sudden shock” that disrupted their day-to-day lives. To gain more insight into the role of social media as an information channel during the early stages of COVID-19, we study the Twitter conversations from that time period.

We investigate two modulators of COVID-19 information diffusion: the social status of the tweet's author (whether they were a Health Expert or an Influencer) and the novelty of the information contained in the tweets. Twitter's platform affords relationship asymmetry, and social status is often a key determiner of information diffusion. Hence, it is interesting to investigate if some public intellectuals selectively amplified certain types of COVID-19 information. Above and beyond the characteristics of the messenger, the quality of the message is also a key factor that determines the speed of diffusion. Information varies in its quality, and it carries the most value when it's unique and timely. Hence, we investigate the role of the novelty of the message in controlling the speed of diffusion of COVID-19 discourse. Informational novelty has been previously shown to be a key factor determining the propagation of online news^12^. We hypothesize it to play a crucial role in determining the spread of information during the early stages of COVID-19 also.

We study the impact of social status and novelty on information diffusion by stratifying them along five discussion themes or social dimensions—health, political, travel, economic, or personal information (see SI for more details). These dimensions capture an axis of a significant impact of the pandemic and were determined based on their prominence in COVID-19 discourse. We study health and travel information since access and dissemination of both these types of information were crucial, especially during the pandemic’s early stages. There were several competing theories, and hence uncertainty regarding the virality of COVID-19^3,8^. The travel regulations imposed by several countries further caused anxiety related to travel over and above the underlying concerns regarding the spread of the virus^13^. The variety of information available on Twitter, coupled with the presence of many health and travel experts (including government officials) on the platform, made it a preferred destination to seek and disseminate health and travel-related information. Third, we study online political discourse during COVID-19 since there were several contentious and polarizing aspects of the pandemic early on, such as lock-downs, wearing masks, and social distancing. For example, most Republicans were against such regulations in the USA, whereas the Democrats favored them^14,15^. On a similar theme, we next studied another macro-level impact of the pandemic—its economic impact. The economic upshot of COVID-19 can not be understated as many businesses closed while others downsized. There was a global spike in unemployment, and financial hardship was pervasive. Finally, we also measure the micro-level impact of the pandemic by studying the personal narratives shared by people online. The quarantine and stay-at-home orders confined millions of people to their homes. Without a doubt, that took a psychological toll on people worldwide. And social media was their ultimate refuge.

## Results

We analyze a randomly selected set of 8 million English language tweets posted between January 23, 2020, and June 22, 2020, that contained at least one COVID-19 related keyword (see SI for collection details). Using machine learning classifiers that employ active learning and ensemble learning, we classify each tweet into the five dominant themes or topics of discussion: Politics, Health, Economy, Travel, and Personal. About 75% of our sample tweets fall into at least one of the above five categories. A single tweet can potentially be classified into more than one discussion topic. For instance, around 12% of tweets were cross-classified into two categories. Additionally, we classify Twitter users into influencers and health experts. Influencers are defined as users with at least 5000 followers and whose accounts have been *verified* by Twitter. Influencers posted about 14% of the tweets in our dataset. Our definition of health experts includes doctors, nurses, health journalists, medical researchers, health ministers, epidemiologists, etc. They were identified via a manual annotation procedure based on their Twitter biographies combined with a machine learning classifier (see SI for more information). About 8% of the tweets in our dataset were posted by health experts. Next, we compute the novelty of each tweet by comparing the topic distribution of its words with the topic distribution of words in all the tweets within the previous 1-day, 3-day, or a 7-day time window. We employ the procedure used by^12,16^ to compute the novelty of tweets (see Methods and SI for more details). We also control for several variables such as the length of the tweet in characters, the number of punctuation marks, and the number of capitalized words in the tweet to adjust for various characteristics of a tweet. Finally, we estimate Poisson regression models using the number of retweets as the outcome variable (see Methods and SI for the model specifications).

**Figure 1:**
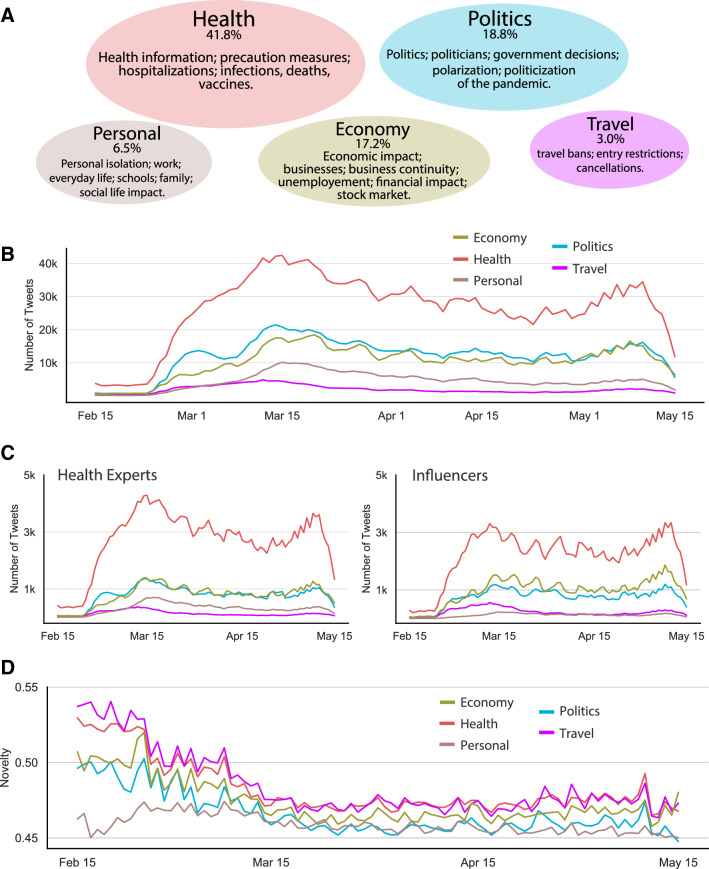
(**A**) The composition of the various online discussion themes. (**B**) Daily count of total tweets (3-day moving average) per social discussion theme. (**C**) Daily number of tweets posted by Health Experts and Influencers on the 5 discussion themes. (**D**) 3-day moving average of the 1-day novelty in the content of tweets in each discussion theme

Figure 1 summarizes our data and the key variables used in our study. As expected, more than one-third of the tweets are health-related, and the dominance of this discussion theme is steady across time, followed by tweets talking about politics (18.8%), economy (17.2%), personal impact (6.5%), and travel (3%). At the very beginning of the pandemic, people discussed travel information more than personal matters; however, later in March and early April 2020, tweets regarding the personal impact began to circulate as much. Though politics was the second most prominent topic of discussion throughout, the gap between the politics and economy-related tweets shrunk towards the start of the summer (Fig. 1B). This hints at the continual economic disruption due to COVID-19 and its increasing prevalence in social media conversations. Health-related information also dominated the thoughts of health experts and influencers, though influencers also communicated a significant amount of politics-related information (Fig. 1C). Travel and health-related discourse on Twitter contained the most informational novelty in the initial stages. The difference between novel information carried by the various categories and the overall informational novelty, though, narrowed over time as more people were exposed to that information (Fig. 1D).

**Figure 2:**
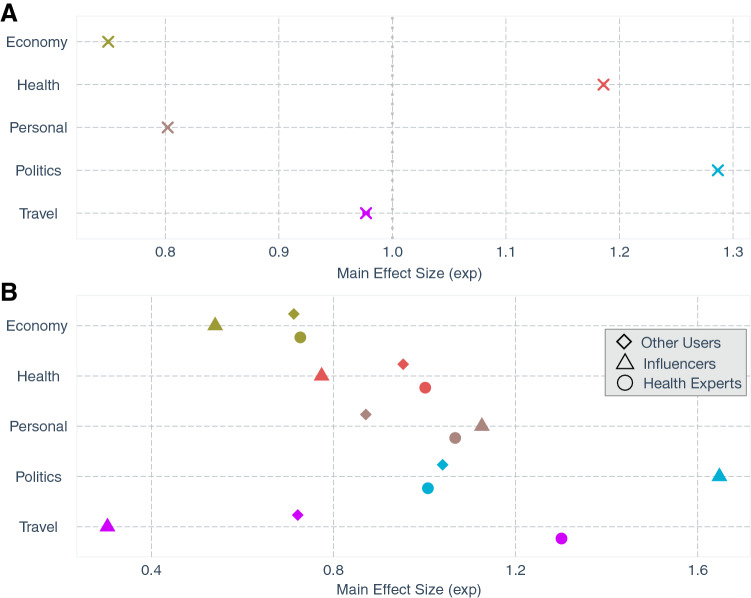
(**A**) The estimated effect sizes (Poisson regression Model 1 in Methods) for information propagated along each discussion theme. For example, the effect size for “politics” being 1.286 means that tweets that fall into the politics discussion theme are retweeted 28.6% more than tweets that do not belong to that dimension. The 95% CIs are smaller than the point estimate symbols. (**B**) The effect (Poisson regression Model 2 in Methods) of the social status of the author (health experts vs influencers vs. other users) on the propagation of the five different themes. For example, tweets about politics were retweeted more when authors were influencers compared to health experts or other twitter users, after controlling for the social status. The 95% CIs are smaller than the point estimate symbols. Note that the coefficients are exponentiated, so a value less than 1 indicates a negative coefficient

Our empirical analyses highlight the differential role of various discussion themes in driving the information propagation on Twitter during the initial stages of the COVID-19 pandemic. First, we observe that political and health-related information was shared more than economy or travel-related information (Fig. 2A). More precisely, health-related information was retweeted around 19% more than other categories, while tweets sharing political content propagated approximately 29% more than tweets from other categories. One expects health information to be shared more widely, but it is a little surprising to see a wider spread of political information during the pandemic. After examining the data, it turns out that the stringent health containment measures adopted globally, e.g., face masks, closures, travel bans, and social distancing, were highly polarizing and gained much attention online. Polarizing content, in general, is known to be retweeted more^17^ and there is evidence of political polarization getting worse during COVID-19, partially attributable to the media’s problematic coverage of the pandemic^18^. All these factors coupled with people’s distrust in traditional media outlets increased their attention towards social media for information updates, thereby boosting the spread of such content. On the other hand, economy-related information, and personal narratives were shared considerably less (about 25% and 20% respectively) than other themes (Fig. 2A). This is potentially due to the gloomy nature of that information and since there were other channels to acquire that information, e.g., government websites, TV, or other local municipal announcements.

Next, we quantify the impact of the messenger on the spread of information. That is, whether the author of the tweet was a health expert, a social media influencer, or none of these. Figure 2B shows that influencers were the most prominent spreaders of political tweets, compared to health experts or other users. This may be attributed to the tendency of such individuals to engage deeper with critical socio-political discussions of the times befitting their social stature. People trusted and re-shared information disseminated by health experts on health-related information (Fig. 2B)—an evidence for belief in science. This also corroborates the empirical findings regarding science-based and fact-checked tweets garnering more engagement than simple facts or opinions during COVID-19^19^. Interestingly, people were engaged more with personal narratives of health experts and influencers (Fig. 2B), due to the authenticity and compassion of such shared experiences. Since the early stages of the pandemic, health experts, including nurses and doctors, shared their struggles and lived experiences while fighting on the front line against the disease. They were frequently touted as heroes, making them the most sought-after personalities all over social media. Similarly, many influencers also raised awareness about several COVID-19 relates issues. However, neither the influencers nor the health experts positively impacted the proliferation of economic information, but health experts did enable fast diffusion of travel information (Fig. 2B).

**Figure 3:**
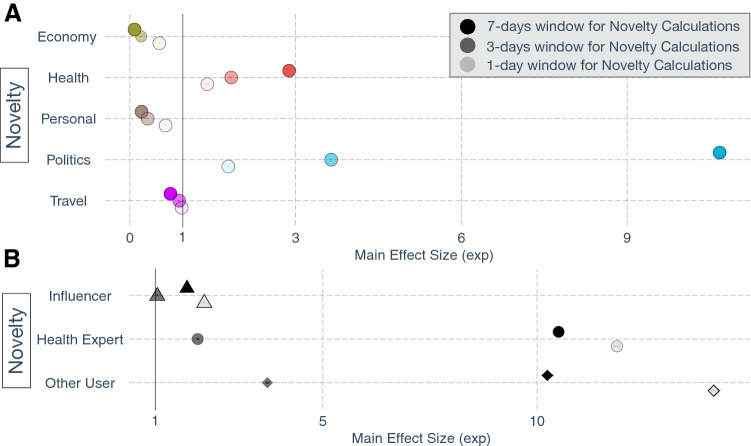
(**A**) The estimated effect sizes (Poisson regression Model 3 in Methods) for the impact of informational novelty on spreading information along each of the five discussion themes. For example, increase in the novelty helped spread political tweets the most. The 95% CIs are smaller than the point estimate symbols hence might not be fully visible. (**B**) The estimated effect sizes (Poisson regression Model 4 in Methods) along with the 95% CIs measuring the disparate impact of propagation of novel information by health experts, influencers, or other twitter users. Novel tweets propagated the farthest when they were authored by health experts compared to influencers or other users. Note that the coefficients are exponentiated, so a value less than 1 indicates a negative coefficient

Finally, we dissect the impact of the novelty of information on its propagation. The impact of novelty on retweets depends on the granularity at which novelty is measured. So, we measured the novelty of tweets by comparing them to the tweets in the previous one, three, or seven days. This accounts for the varying decay rates for different types of information. For example, travel information as well as health information, e.g., latest health and safety guidelines, got outdated every day due to the infusion of much new information. However, other topical news was getting refreshed at a slower rate.

Novel information, in general, spread faster (Fig. 3A). Content novelty modulated the proliferation of health and political information, further increasing their reach. Novelty accelerated the spread of health and political news due to the broad appeal and applicability of such communications. Such tweets often contained preventive health information, announcements, or opinions on polarizing political issues. For example, some highly novel tweets in the early stages of the pandemic constituted information regarding developing a vaccine or genome sequencing of the novel coronavirus. The fast diffusion of these conversations was also partly due to them being discussed ad infinitum on Twitter and other social media, leading to disseminating much repetitive information. So, these topics’ importance coupled with their increased availability leads to the uniqueness of the information being the driving factor in its spread.

On the other hand, novelty impeded the spread of the economy and personal information shared by users. Investigating the underlying tweets, the main reason for this was the nature of such information, which was not critical enough to broadcast to a broader audience. Interestingly, we can also notice (Fig. 3A) that the positive coefficients became more positive and the negative ones became more negative; that is, the absolute value of the coefficients increased with an increase in the size of the rolling reference window for computing novelty. This suggests temporal aggregation of information, which made the relative informational content (and hence the associated novelty) of a tweet a more salient predictor of its spread when compared over wider time windows.

Intriguingly, next, we notice that novel information was propagated more when health experts or the crowd (other users) authored it (Fig. 3B). People preferred to share unique information that they deemed critical to mobilizing due to the pandemic’s nature. They did pay attention to the author’s social status, especially when they were health experts. However, they subdued their propensity to retweet the influencers when it came to novel information due to a potential lack of trust since of them might be paid to advance novel rhetoric or theories pertaining to the spread of COVID-19.

Zooming into the novelty results further (Table S16 in the Appendix), we see a U-shaped quadratic relationship between the informational novelty (stratified into quintiles) and the number of retweets of a tweet. Tweets with average novelty were propagated the least. Tweets with high novelty spread faster when they contained political, health, or personal information, due to the immediacy of the government response or the need to spread important health information. Further, during the early stages of COVID-19, highly novel personal anecdotes (e.g., related to symptoms or diagnoses) often expressed nuanced emotions which resonated with others. Many of them were also touching and narrated the pandemic’s disparate impact at the micro-level. Hence they also propagated faster. Conversely, economic and travel information was shared more when it was less novel. Based on the data, we notice it is due to the hesitation in propagating highly novel job-related or travel restrictions-related information since such information was continuously evolving, and partly due to the potential of such novel information, if incorrect, to create chaos among people.

## Discussion

Undoubtedly, the COVID-19 pandemic has had a far-reaching impact on our society and economy. It will have long-term ramifications on every facet of our lives, and we are still grappling with its short-term effects. Twitter has played an important and timely role in accessing and disseminating information at different stages of this pandemic. In this paper, we empirically analyzed the role played by Twitter in the propagation of information via the lens of five key discussion themes—Travel, Health, Personal, Politics, and Economy. We studied how the information on these five dimensions was modulated by the author’s status and the novelty of the information contained in the tweet. Several key findings result from our analyses. Twitter’s social media platform provided a conduit for people to relate to others’ personal experiences with COVID-19, receive updates on health-related information, and engage with political opinions. The spread of news on these topics was further accelerated by the novelty of the content of the tweets, e.g., users engaged more with novel information when health experts shared it. Influencers were, however, effective in spreading political news. We observed a U-shaped relationship between the informational novelty and the number of retweets. Tweets with average novelty spread the least. Tweets with high novelty propagated the most, primarily when they discussed political, health, or personal information, perhaps owing to the immediacy to mobilize this information. On the other hand, economic, and travel-related information spread most when it was less novel. People resisted sharing such information before it was duly verified as it was anxiety-provoking.

Our study does have limitations. First, it only captures the “online” impact of the pandemic and cannot tap into the offline conversations that materialized during the period of our study. Second, our work does not disentangle the dynamics of misinformation. Our scientific objective in conducting this study was to analyze the diffusion of novel information holistically and how it got modulated by health experts and influencers in the early stages of the pandemic. Hence, we did not analyze misinformation separately. That said, our back-of-the-envelope calculation suggests that there were about ~ 17% tweets containing false information in our data sample, which is similar to the number reported in other COVID-19 studies^9^. It is an exciting avenue for future research to understand the dynamics of diffusion of COVID-19 misinformation.

## Material and methods

We analyze a randomly selected set of 8.25 million original tweets posted between January 23, 2020, and June 22, 2020, that contained at least one COVID-19 related keyword (ncov2019, nCov-2019, COVID-19, covid2019, nCov19, 2019nCoV, nCov2019, Wuhan virus, COVID19, Coronavirus, covid19, covid-2019, covid2019, 2019-nCov, nCov, SARS-CoV-2 and COVID-19). The collected data comprised a total of 19 million Twitter messages, out of which we filtered out retweets, quotes, and non-English language tweets.

### Text classification

Using machine learning classifiers that utilized active learning (with the help of 9 senior-year and master students that manually annotated a gold standard corpus of more than 4000 tweets) and ensemble learning, we classify each tweet into the five dominant themes or topics of discussion: Politics, Health, Economy, Travel, and Personal. For each social dimension, we built 6 classifiers where each one was trained on a different set of text features, and the results were aggregated in an ensemble learning framework. The accuracy of the resulting one-vs-all classifiers were 89.9%, 83.6%, 99.0%, 89.0%, and 89.3% for Politics, Health, Travel, Personal, and Economy respectively. About 75% of our classified tweets fall into at least one of the above five categories. A single tweet can potentially be classified into more than one discussion topic. For instance, ~ 12% of tweets were cross-classified into two categories. Additionally, we classify Twitter users into influencers and health experts. Influencers are defined as users with at least 5000 followers and whose accounts have been *verified* by Twitter. Influencers posted about 14% of the tweets in our dataset. Our definition of health experts includes doctors, nurses, health journalists, medical researchers, health ministers, epidemiologists, etc. Health experts were identified via a manual annotation procedure followed by a machine learning classifier. Three annotators annotated a total of 500 Twitter account biographies which were used to train a machine learning classifier, achieving 85% accuracy and 96.5% recall. About 8% of the tweets in our dataset were posted by health experts. A Twitter user can both be classified as influencer and health expert. These users account for 0.1% of the tweets, and 0.3% of the users in our corpus.

### Novelty calculation

We calculate the novelty of information contained in each tweet. The novelty of a tweet’s content is computed by comparing the topic distribution of words in the tweet with the topic distribution of words in all the tweets within the previous 1, 3, and 7 days time window. We employ the procedure used by^12,16^ to compute the novelty of tweets which calculates the information uniqueness by computing the cosine similarity of the topic distributions.

### Regression specifications

Finally, we estimate Poisson regression models using the number of retweets $Y_i$ as the outcome variable. We selected the Poisson regression framework as it is suitable for count data with a long tail. A number of controls were used such as the length of the tweet in characters, the number of punctuation marks, and the number of capitalized words in the tweet to adjust for various characteristics of a tweet. We replicate our analysis using the number of “likes” as the dependent variable. Those results are broadly similar to the ones reported in the manuscript and can be found in SI. We consider the following model specifications: **Model 1**: $Y_{i} = \beta _{0} + \text{theme}_{i} + \text{controls}_i + \epsilon _{i}$, **Model 2**: $Y_{i} = \beta _{0} + \text{author status}_{i} + \text{theme}_{i} + \text{theme}_{i} \times \text{author status}_{i} + \text{controls}_i + \epsilon _{i}$, **Model 3**: $Y_{i} = \beta _{0} + \text{novelty}_{i} + \text{theme}_{i} \times \text{novelty}_{i} + \text{controls}_i + \epsilon _{i}$ and **Model 4**: $Y_{i} = \beta _{0} +\text{author status}_{i} + \text{novelty}_{i} + \text{novelty}_{i} \times \text{author status}_{i} + \text{controls}_i + \epsilon _{i}$.

## Supplementary Information

Supplementary Information.

## Data Availability

Data and Code are available at a GitHub repository: https://github.com/aphoti01/covid19-social-status-and-novelty.
