# Supplementary material for: Social status and novelty drove the spread of online information during the early stages of COVID-19
Source: Sci Rep. 2021 Oct 11;11:20098. doi: 10.1038/s41598-021-99060-y (PMC8505518; doi:10.1038/s41598-021-99060-y)
Supplement: Supplementary file 1 — Supplementary Information. [file 41598_2021_99060_MOESM1_ESM.pdf]

Supplementary Materials for: Social Status and Novelty Drove the Spread of Online Information During the Early Stages of COVID-19

Antonis Photiou,[1] Christos Nicolaides,[1,3*] Paramveer Dhillon[2,3*]

[1]School of Econ. & Management, University of Cyprus, Nicosia, Cyprus, 2109
[2]School of Information, University of Michigan, Ann Arbor MI, USA 48109
[3]MIT Initiative on the Digital Economy, Cambridge MA, USA 02142
[*]To whom correspondence should be addressed;
Email: nicolaides.christos@ucy.ac.cy & dhillonp@umich.edu.

September 24, 2021

Contents

S1 Data **S3**

S1.1 Tweets S3

S1.2 Twitter users S3

S2 Social Dimensions **S4**

S2.1 Text Classification S7

S2.1.1 Feature Engineering S7

S2.1.2 Model Building S9

S2.1.3 Active Learning S12

S3 Quantifying Novelty **S12**

S4 Categorizing Twitter users **S13**

S5 Regression Analysis **S15**

S5.1 Model specifications S15

S6 Regression Tables **S17**

S6.1 Response Variable: Retweet count S17

S6.1.1 Response Variable: Is retweeted S24

S6.1.2 Quadratic relationship between Novelty and Retweet countS29

S7 Sample of highly reshared tweets **S30**

S1 Data

S1.1 Tweets

As our task was to explore the online information spread during the COVID-19 pandemic, we collected Twitter data containing virus related information. Twitter was selected as the main data source for this study, as it provides it's data timely through APIs in contrast to the other popular social media platforms. The brand monitoring site Brand24 (https://brand24.com) was used, which collects data from social media, news, blogs, videos, forums, reviews and more. Brand24 continuously seeks for new and existing mentions across the web about the used keywords. The first collection started on March 11 2020, although the collected data date back to January 23 2020. The keywords used for the collection of data are: 'n_cov2019', 'nCov-2019', 'COVID-19', 'covid2019', 'nCov19', '2019nCoV', 'nCov2019', 'Wuhan virus', 'COVID19', 'Coronavirus', 'covid_19', 'covid-2019', 'covid_2019', '2019-nCov', 'nCov', 'SARS-CoV-2' and 'COVID-19'.

Brand24 provides the tweet ids where the mentions match the specified keywords. The tweet ids are separated from the rest of the mentions, and the Twitter API was used for the collection of the tweet information. Approximately 55% of the tweet ids can be retrieved using the Twitter API (12.17 million out of 19.22 million). The collection is done every 2 days over 8-day windows. Therefore, the collections were overlapping in order to (1) update the impressions (likes, retweets, etc.) and (2) to collect as many tweets as possible due to the fact that they might be removed. Out of the total of 9.5 million virus-related tweets, retweets and quotes collected, only 8.25 million English language tweets were used for the analysis.

S1.2 Twitter users

Twitter users were then separated from the tweets in order to (1) avoid having redundant information in the tweets dataset and (2) to have the most recent information available in relation to user characteristics such as the number of followers and the total number of retweets. The location of each user is inferred using the method proposed by Compton et al. (2014) [4], which infers a user's location by examining their connections' locations. The algorithm was capable of inferring location for more than 100 million users at a median error of 6.38 km and geotagging around 80% of public tweets. Using this method we were able to geotag 3.24 million tweets or 51.42% of our corpus. Furthermore, the gender, age group and, whether the account belongs to an organization account or not, are inferred using the m3inference model. M3 is a deep learning system for demographic inference that was trained on a massive Twitter dataset ([10]). M3 provides a full model which uses vision (profile picture) and text (name and bio) inputs and, a text-only model to make the predictions. The full model was used for the majority of the twitter users. The text model was used on just 3% of the users where the profile picture was not compatible for the model. The

	Followers	Friends	Listed	Statuses count	Favourites count
Mean	10433.562	1731.109	89.476	23141.422	17733.148
St. Deviation	263844.772	7495.667	877.456	59524.206	38798.634
Minimum	0	0	0	0	0
1st Quartile	282	317	2	3094	1281
2nd Quartile	798	698	11	8319	4894
3rd Quartile	2465	1578	47	22783	16928
Maximum	82441111	1584592	379695	30441279	2045776

Table S1: User level summary statistics

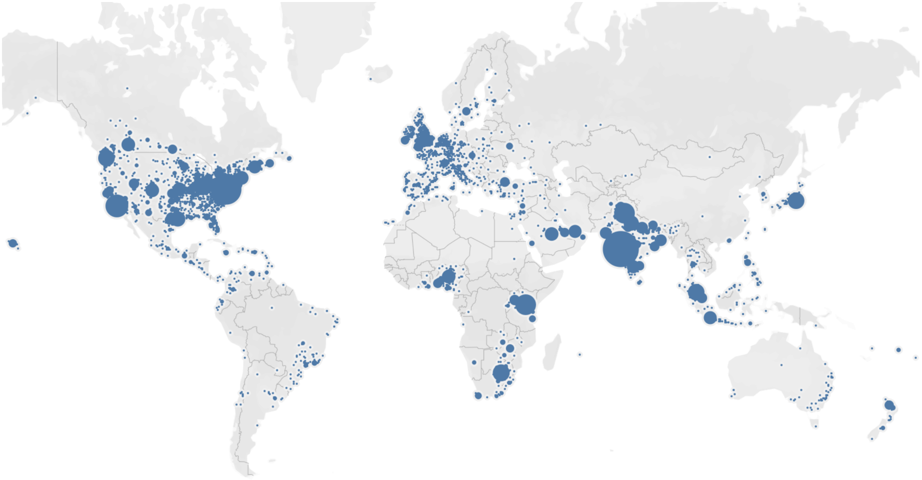

Figure S1: World map with size of conversation by city. The size of the bubble is proportional to the number of tweets in the area. Maps were generated in the software Tableau.

tweets were posted by 1.08 million users of which 66.9% were males, 72.3% were personal accounts, and 5.5% of the accounts were verified. The m3inference models helped us infer the age group of the users; 36.2% were under 19, 13.3% were between 19 and 29, 33% were between 29 and 39, and 17.4% over 39 years old.

S2 Social Dimensions

One of our main goals is to analyze the social dimensions in Twitter conversations, therefore we need to identify the main topics of the discussions in the tweets. To identify these dimensions, initially, 3 researchers manually browsed through a subset of the tweet corpus to form the broader themes. Five high level social dimensions were identified: Politics, Health information, Travel in-

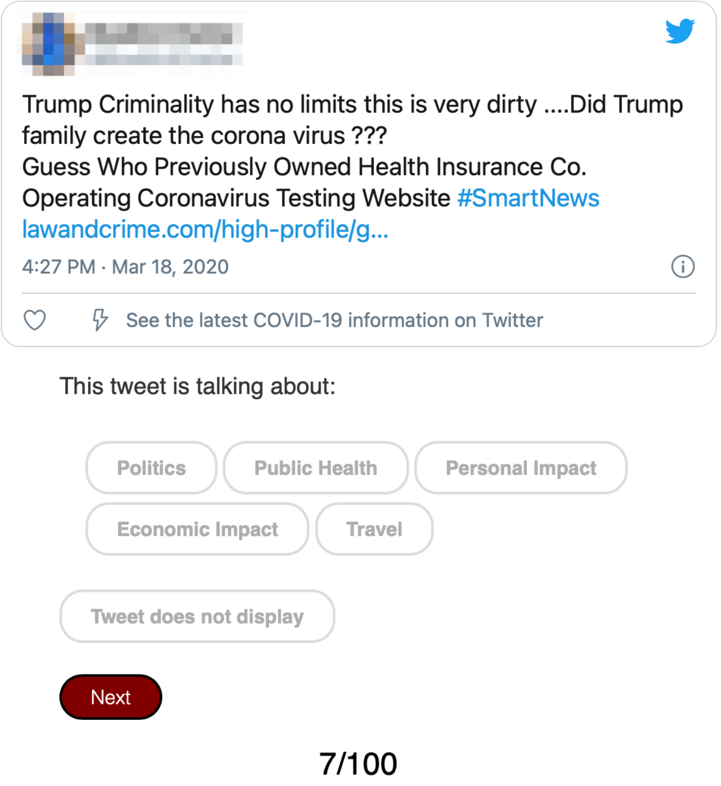

Figure S2: Screenshot of the survey website that was used to annotate the tweets. Annotators were given clear instructions on selecting one, or more categories or if the tweet does not belong to any of the five categories, not to select any category and continue to the next task.

formation, Personal impact and Economic impact. 'Politics' dimension includes discussions about politicians, government decisions, etc., including the writers' personal opinion and media news. The category 'Health Information' contains tweets that talk about precautions, measures, infections, deaths, vaccines, and anything related to health. 'Travel Information' includes information about traveling, travel bans, restrictions, cancellations, etc. 'Personal Impact' tweets contain the writer's impact due to the virus, including information about isolation, work, everyday life, school, family and friends. Finally, tweets belonging to the 'Economic Impact' category contain information about business, business continuity, distance working, firings, financial impact, stock market, etc.

We study health and travel information since access and dissemination of both these types of information were crucial, especially during the pandemic's early stages. There were several competing theories, and hence uncertainty regarding the virality of COVID-19 [5, 8]. The travel regulations imposed by several countries further caused anxiety related to travel over and above the underlying concerns regarding the spread of the virus [3]. The variety of information available on Twitter, coupled with the presence of many health and

Category	Sample keywords	% of total
Politics	Trump, government, lock-down	18.78
Health information	health, spread, CDC, WHO	41.84
Travel information	travel, flight, cancellation	2.98
Personal impact	isolation, personal, love	6.53
Economic impact	crisis, market, business	17.23

Table S2: Proportion of tweets in each social dimension

travel experts (including government officials) on the platform, made it a preferred destination to seek and disseminate health and travel-related information. Third, we study online political discourse during COVID-19 since there were several contentious and polarizing aspects of the pandemic early on, such as lock-downs, wearing masks, and social distancing. For example, most Republicans were against such regulations in the USA, whereas the Democrats favored them. On a similar theme, we next studied another macro-level impact of the pandemic—its economic impact. The economic upshot of COVID-19 can not be understated as many businesses closed while others downsized. There was a global spike in unemployment, and financial hardship was pervasive. Finally, we also measure the micro-level impact of the pandemic by studying the personal narratives shared by people online.The quarantine and stay-at-home orders confined millions of people to their homes. Without a doubt, that took a psychological toll on people worldwide. And social media was their ultimate refuge.

Due to the large volume of tweets, manual classification to the five categories was not feasible. Consequently, a subset of human-annotated tweets was used to build machine learning models that would accommodate classifying the remaining data. A subset of tweets needed to be manually labeled with the five social dimensions, on which the machine learning models would be trained. As part of this step, we created a survey website for annotators to classify the tweets (see Figure S2). The website fetches a random tweet at a time from the corpus and the participant has to classify the tweet into none, one or, more than one of the 5 pre-specified categories. To ensure the validity of the results and to filter out potential bots answering the survey, a small fraction of the tweets were used to test the participant. The label of the testing tweets was prespecified by the researchers and in case the participant failed to classify the test tweets correctly, the responses were not recorded. The survey was given to University of Cyprus and University of Edinburgh undergraduate and postgraduate students at first stage. The annotators were given brief descriptions of the five categories and were allowed to have five warm up tweets. In total, nine annotators were employed and the inter-annotator agreement was calculated using the statistical measure Fleiss' Kappa. The nine annotators were split into 3 teams where each team classified a subset of 100 tweets. The Fleiss' kappa (κ) was calculated 0.85, 0.81 and 0.83 for team one, two and three respectively, whereas the agreement between two annotators ranges from 84% to 91%.

	Annotator 1	Annotator 2
Annotator 2	88%	
Annotator 3	90%	87%

	Annotator 4	Annotator 5
Annotator 5	86%	
Annotator 6	84%	85%

S2.1 Text Classification

The next step involved building machine learning classifiers based on the annotated data that would help us assign social dimensions to the entire tweet corpus. Although, annotated data are not sufficient for building a machine learning classifier since raw text cannot be directly used as an input. Textual data is a form of unstructured data that does not come in a predefined form or structure. Therefore, crucial steps should be taken to make the data suitable for the analysis. First, text data should be cleaned to remove the noise. Due to a large number of users coming from different backgrounds and habits, Twitter data contain a lot of noise in the form of emojis, emoticons, slang language, URLs, and punctuation. Worthless information such as stop-words should be removed and other information such as emojis and symbols should be annotated for later use. The python libraries ekphrasis and nltk were used to perform the data cleaning. Examples of the textual data before and after the cleaning are presented in table S4.

The final step of data pre-processing is generating the root form of inflected words. Stemming and lemmatization are two methods of dealing with this task. Porter's stemming algorithm consists of five rule-based phases that reduce the word. On the other hand, lemmatization does a full morphological analysis to accurately identify the lemma for each word. Lemmatization was selected for this analysis as a full morphological breakdown of a word was providing more accurate results.

S2.1.1 Feature Engineering

Next, the textual data was transformed into numerical feature vectors that would serve as model inputs. There are different approaches to transforming raw text into feature vectors. Five different text transformation methods were used, resulting to 6 different datasets. Due to the complexity of the task, the different feature engineering techniques were used together to obtain better results. The methods are summarized below:

- **Count vectors**: The simplest approach of representing text with numerical vectors. Count Vector is a matrix notation of the dataset in which every row represents a tweet from the set, every column represents a term from the corpus, and every cell represents the frequency count of a par-

	Annotator 7	Annotator 8
Annotator 8	91%	
Annotator 9	87%	86%

Table S3: Agreement between annotators on classifying tweets in social dimensions

Before	After
Good morning everyone have a great day	good morning everyone great day thumb good_morning morning_everyone everyone_great great_day day_thumb
Rural America Could Be the Region Hardest Hit by the COVID-19 Outbreak https://t.co/4uHTemPo5s	rural america could region hardest hit covid ¡number¿ outbreak <url> rural_america america_could could_region region_hardest hardest_hit hit_covid covid_<number> <number>_outbreak outbreak_<url>

Table S4: Text pre-processing

ticular term in a particular tweet.

- **TF-IDF Vectors**: The TF-IDF score exemplifies the relative significance of a term in the tweet and the entire corpus. TF-IDF score is comprised by two terms: the first computes the normalized Term Frequency (TF), the second term is the Inverse Document Frequency (IDF), computed as the logarithm of the number of the documents in the corpus divided by the number of documents where the specific term appears. Bigram-level TF-IDF vectors were used, as they were found to have better predictive ability than the word- and character-level.

$$tfidf(t,d,D) = tf(t,d) * idf(t,D) \tag{1}$$

$$tf(t,d) = f_{t,d} / \sum_{t' \epsilon d} f_{t',d} \tag{2}$$

$$idf(t,D) = log(n/N_t) \tag{3}$$

where term t occurs in document d which belongs to corpus D.

- **Word Embeddings**: Word embeddings are learned representations of words in a predefined vector space. Similar words tend to have alike word embeddings and that makes the approach suitable for vectorizing text for machine learning use. Two-word embedding algorithms were used; word2vec and doc2vec. Both models were trained on the entire corpus of tweets collected.

Original	Lemmatization	Stemming
With guidelines changing on a daily basis, make sure you're keeping up to date with your work emails and other communications. #SCASproud #covid19	guideline change daily basis make sure ' keep date work email communication sca sproud covid	guidelin chang daili basi make sure ' keep date work email commun sca sproud covid
Coronavirus: Hydroxychloroquine evidence for treating Covid-19 is weak - Vox https://t.co/gjdFw3n	coronavirus hydroxychloroquine evidence treat covid <number> weak vox <url>	coronaviru hydroxychloroquin evid treat covid <number> weak vox <url>

Table S5: Stemming vs. Lemmatization for generating the root of the word

- **Word2vec**: Google developed this two-layer neural network that processes text by vectorizing words. Its input is a text corpus and its output is a set of vectors: feature vectors that represent words in that corpus. [7]
- **Doc2vec**: Doc2vec is an extension of word2vec which vectorizes documents instead of words. It was developed by Le and Mikolov (2014) [6].

- **Topic modeling**: An unsupervised technique that is used to group words into topics from a large corpus of text. Latent Dirichlet Allocation (LDA) algorithm for topic modeling was used. LDA is a matrix factorization technique. which represents any corpus as a document-term matrix. [2]
- **Natural Language Processing (NLP) features**: Text-based features such as word count, character count, word density, punctuation count, upper case count, and title count. Although NLP features do not perform well in text classification tasks, they help improve other classification models.

S2.1.2 Model Building

One vs. rest machine learning models were preferred to multi-label classification due to more accurate results. Therefore, predicting each category was a separate classification task. Six data-sets were prepared, one for each of the feature engineering methods described above. For the prediction of each category, six classifiers were trained, one for each of the feature sets. Training different algorithms on each feature set, and aggregating the results, serves to accomplish more accurate predictions using a meta classifier. Four machine learning algorithms were used, a Support Vector Classifier and three variations of Logistic

Regression; with L1 regularization, with L2 regularization and, with Stochastic Gradient Descent (SGD) training.

A binary support vector classifier aims to find a hyperplane in an N-dimensional space, where N is the number of features, that separates the two classes with the maximum margin between the two. Two parameters of the support vector classifier were tuned, C and class weight. C parameter adds a penalty for each misclassified observation. When C is small, the penalty is low so a decision boundary in the training has a large margin. Class weight penalizes the two classes differently in order to put more emphasis on one class or, address unbalanced dataset issues which was the case in this task.

Also known as Logit, logistic regression estimates the probabilities of the possible responses which are modeled using a logistic function. Due to the high dimensions of the engineered datasets, a regularization method was used for penalizing the coefficients. Lasso regression employs L1 regularization uses the parameter λ to penalize coefficients, which forces the less important coefficients to zero. Ridge regression uses a similar regularization, L2, which penalizes the coefficients based on λ but they never reach zero. Stochastic Gradient Descent training is an optimization technique that is used to train the models. The λ parameter and class weight were tuned for the Logistic regression models

For each feature set, the four algorithms were trained and the best one was selected. The model selection was done using the 10-fold cross-validation f1 test score. The best model was then hyper-parameter tuned and stored. By the end of this procedure, six classifiers are trained, one for each of the engineered datasets.

Tweet	Social Dimension
COVID-19: Lloyd's to stress test the market and corporation resilience. @LloydsBank has announced measures that aim to provide reassurance to the market. https://t.co/ro8dXrrcbi#Lloyd's #Coronavirus #Insurance #Reinsurance	Economy
The Trump Organization is seeking U.K. and Irish bailout money to help cover wages for bartenders, bagpipers and other employees furloughed from its European golf properties because of the coronavirus lockdown.https://t.co/5eAABuQKMl	Economy
As #COVID19 sweeps South Korea, a South Korean socialist says, 'Our government protects profit, not people' #coronavirus https://t.co/jT2GNBVg61https://t.co/nY1VPfyLVc	Politics
@vp So you are saying the next 60,000 Americans who die are NOT as important as the First 60,000. That they wont need HELP getting supplies from medical teams. As businesses open #Covid19 resurges they are going to need Guidance. What, you dont WANT to give ANY?? #TrumpGenocide https://t.co/FzkMvyOaL4	Politics
Alcohol can have a range of harmful effects on the body, which can diminish a person's immune response and increase risk of becoming more vulnerable to COVID-19 https://t.co/vTt9Ol2orR	Health
In view of the challenges being faced, particularly by the large cities, it was discussed to augment testing as well as the number of beds and services to effectively handle the peak surge of daily cases: Prime Minister's Office (PMO) #COVID19	Health
$1200 will not pay my rent let alone the rest of my bills. What about you?	Personal
Muting your favorite WhatsApp group hurts, but that's another way how Corona has hit me #Corona	Personal
British Airways says it is operating special flights from 11 airports to take back thousands of UK nationals stranded in India due to #coronavirus outbreak #coronavirusinindia	Travel
The State Department issued a Level 4 travel advisory, telling Americans they should not travel to the Lombardy and Veneto regions in northern Italy due to the coronavirus outbreak. https://t.co/RwkjTA3Arj	Travel

Table S6: Sample tweets from each social dimension

S2.1.3 Active Learning

Due to the reason that the amount of data annotated was very low in comparison to the entire tweet corpus, the method of active learning was used to accelerate the optimization of the classifiers. Active learning is a process to prioritize the data to be labeled to have a higher impact on the training of the model. First, the machine learning model is trained on a small subset of the data. Then, the unseen observations are assigned a priority score with the task of assigning higher priority to observations that the classifier was more unsure of the category. Applications of active learning use the least confidence, margin sampling, or entropy among others to measure priority. Although, for this study, the presence of six classifiers for each task was perceived as suitable for prioritizing observations.

The built classifiers are applied to the unseen data and the observations where three of the classifiers predicted that a tweet belongs to a specific category and the remaining three predicted that it doesn't, are separated for human classification. A subset of 100 tweets from each social dimension was then given to human annotators to manually classify them. Then, the models were trained again and the process was repeated until no significant improvement was found in the model performances. The process was repeated 4 times and the final human-annotated dataset consisted of 4036 tweets. The classifiers assigned at least one social dimension to 75% of the tweets in our corpus.

The performance metrics for each of the five categories are presented in the following table:

Category	Accuracy	Precision	Recall
Politics	0.8991	0.8214	0.6764
Health information	0.8359	0.8703	0.7709
Travel information	0.9798	0.8936	0.7924
Personal impact	0.8902	0.8134	0.6774
Economic impact	0.8928	0.8289	0.7727

Table S7: Classifier metrics

S3 Quantifying Novelty

The next step in our analysis was to measure the novelty of information contained in a tweet.

In order to measure the tweet-level novelty of information, we compared each tweet with the information included in preceding tweets. The topic distribution of a tweet i is compared to the topic distribution of all the tweets posted in different time windows preceding the tweet. Cosine similarity was used to measure the distance between the two vectors. To obtain the topic vectors we used the Doc2Vec model which was trained on 8.25 million COVID-

19 related tweets. Doc2Vec was preferred to other numeric representations of text documents, as documents with similar content but not necessarily the same words tend to have alike word embeddings and that helps to capture information uniqueness. The cosine similarity between each tweet's numerical representation and of all the tweets for the previous 1, 3, and 7 days was computed. The average cosine similarity for each tweet was then normalized between 0 and 1, and novelty was calculated as one minus the normalized average, where higher values of novelty correspond to higher uniqueness of information. The aforementioned approach is also described in the literature as information uniqueness [1, 9]. Additionally, we discretize novelty into fixed sized intervals in order to study the effect of each level. To construct the categorical variable, we break novelty into 5 equal sized bins based on the value of novelty.

$$Similarity(A, B) = cosine(\theta) = \frac{A * B}{||A|| * ||B||} \tag{4}$$

where A, B are the Doc2Vec topic vectors of two tweets.

S4 Categorizing Twitter users

We categorize Twitter users to Influencers, Health experts or other users in an attempt to capture the effect of such users in the outcomes of the analysis. Influencers are defined as users with at least 5000 followers and whose account is verified. By selecting only verified accounts we aim to avoid bots and malicious accounts. Zafar et al. (2015) described an approach of identifying topic experts using Twitter Lists [11]. Although, for the scope of this study only health experts were required. Health experts include doctors, nurses, health-specific journalists, medical researchers and health ministers. In order to identify health experts human annotation was done before employing machine learning. Three annotators annotated a random subset of 500 Twitter users based on their Twitter account bio. Then, users' data were cleaned in the same way as S2.1 and their count vectors were obtained to represent the text. Different machine learning classifiers were build to predict whether a user is a health expert based on their bio. Table S8 shows the performances of the used models. SGD Classifier was the best performing model with 85% accuracy and 96.45% recall implying that 96 out of 100 classified users as health experts were actually health experts. On the final dataset used, we found 43206 (4%) Influencers who produced 14% of the tweets and, 83475 (7.7%) health experts who produced 8% of the tweets.

Model	Accuracy	Precision
Logistic Regression	0.8045	0.9014
Ridge Classifier	0.8359	0.9134
SGD Classifier	0.85	0.9645

Table S8: Classifier metrics - Health experts classification

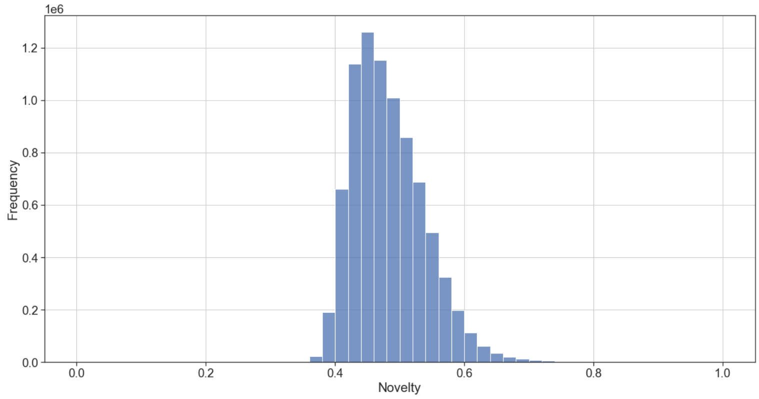

(a) Histogram of Novelty.

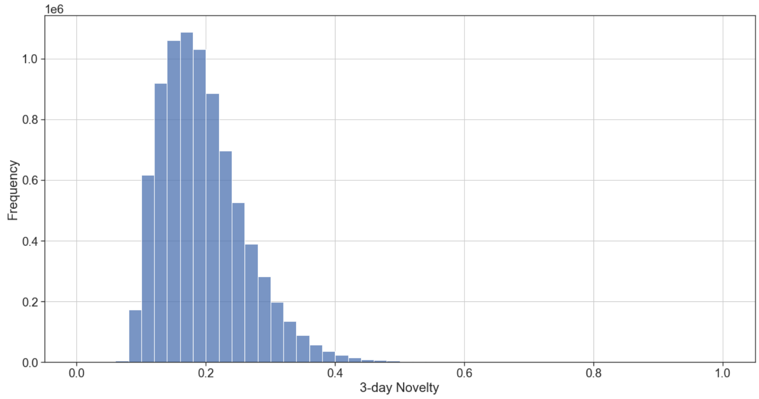

(b) Histogram of 3 day Novelty.

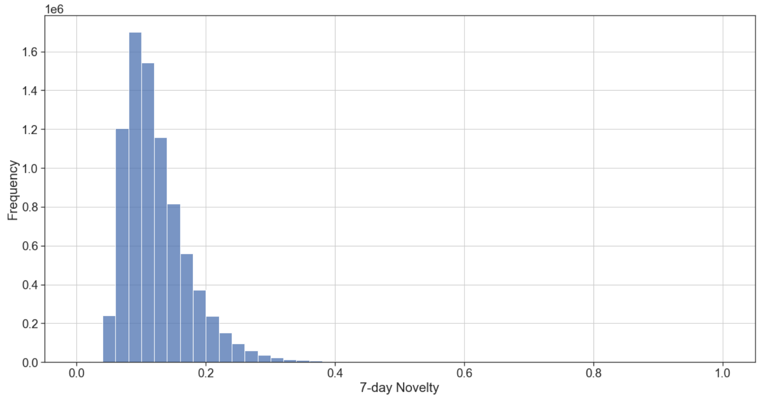

(c) Histogram of 7 day Novelty.

Figure S3: Novelty histograms

S5 Regression Analysis

As our task was to study the the spread of information in the social media platform, we selected the number of retweets and whether the tweet was retweeted or not as the dependent variables. We modelled these variables in a Poisson regression framework in order to estimate the effects of individual-level and society-level dimensions in disseminating information related to the pandemic. Poisson regression is a generalized linear model which assumes the Poisson distribution as the probability distribution of the response variable, therefore establishing it suitable for modelling count responses. Non-robust covariance type and log link function were used for all the regressions.

S5.1 Model specifications

We modelled 4 different model specifications for each response variable, utilizing various collected and calculated features. Influencers and Health Professionals resemble the author status features. In our final dataset, 14% of tweets were posted by Influencers, while 8% of tweets by health experts. We refer to users that do not fall in ether the Influencers or in Health Experts category as "other users". The social dimension features consist of Politics, Health, Personal, Economic, and Travel and were labelled through the text classification framework presented in S2.1. As described above, a tweet may belong to more than one of these social dimensions, having for example 12% of the tweets fall into 2 of the five categories. We control for tweet characteristics, Character Count, Punctuation Count, and Upper Case Word Count, which reduces the likelyhood that the spread of information is driven by those characteristics. Novelty was used both as a continuous and a categorical variable. The continuous variable was used in order to examine the linear and quadratic relationship with the response variable, while the categorical variable was used to investigate the effect of the tails. The three control variables were normalized between 0 and 1 using min-max scaling, so that they are at the same scale. All the used features are described in table S9 and their summary statistics is presented in table S10.

Feature	Description
Politics	Whether the tweet is talking about Politics (1) or not (0)
Health	Whether the tweet is talking about Health (1) or not (0)
Personal	Whether the tweet is talking about Personal matters (1) or not (0)
Economic	Whether the tweet is talking about the Economy (1) or not (0)
Travel	Whether the tweet is talking about Travel (1) or not (0)
Novelty	Tweet-level measure of novelty of information, calculated using tweets posted 24 hours before a tweet
Novelty-3day	Tweet-level measure of novelty of information, calculated using tweets posted 3 days before a tweet
Novelty-7day	Tweet-level measure of novelty of information, calculated using tweets posted 7 days before a tweet
Influencer	Whether the user is verified and has more than 5000 followers (1) or not (0)
Health Professional	Whether the user is a health professional (doctor, health journalist, professor, etc.)
Character Count	Count of characters in the tweet
Punctuation Count	Count of punctuation (!, ", $, %, &, ', (,), *, +, ,, -, ., /, :, ;, =, ?, [,], ^ _, ‘, {, —, }, , •, ') characters in the tweet
Upper Case Word Count	Count of words in the tweet with all upper case letters

Table S9: Tweet-level analysis features description

Statistic	N	Mean	St. Dev.	Min	Pctl(25)	Pctl(75)	Max
Politics	8256385	0.1879	0.3906	0	0	0	1
Health	8256385	0.4185	0.4933	0	0	1	1
Personal	8256385	0.0654	0.2472	0	0	0	1
Travel	8256385	0.0298	0.1701	0	0	0	1
Economic	8256385	0.1723	0.3777	0	0	0	1
is_retweeted	8256385	0.2752	0.4466	0	0	1	1
RetweetCount	8256385	4.3286	212.4015	0	0	1	224512
Novelty	8256385	0.4827	0.0558	0	0.4408	0.5174	1
Novelty_3day	8256385	0.1950	0.0652	0	0.1468	0.2312	1
Novelty_7day	8256385	0.1235	0.0511	0	0.0874	0.1475	1
CharacterCount	8256385	0.1626	0.0794	0	0.0929	0.2336	1
PunctuationCount	8256385	0.0199	0.0115	0	0.0130	0.0260	1
UpperCaseWordCount	8256385	0.0152	0.0246	0	0	0.0215	1

Table S10: Summary statistics of the variables used in the regression specifications.

Next, we present the model specifications for the 4 regressions:

Regression 1 - Estimating the response using the social dimensions as predictors.

$$Y_i = \beta_0 + dimension_i + controls_i + \epsilon_i$$

Regression 2 - Estimating the response using the author's status and its interactions with social dimensions.

$$Y_i = \beta_0 + authorstatus_i + dimension_i + dimension_i \times authorstatus_i + controls + \epsilon_i$$

Regression 3 - Estimating the response using the social dimensions and their interactions with Novelty.

$$Y_i = \beta_0 + novelty_i + dimension_i \times novelty_i + \\ + controls + \epsilon_i$$

Regression 4 - Estimating the response using the author's status and its interactions with novelty.

$$Y_i = authorstatus_i + novelty_i + novelty_i \times authorstatus_i + controls + \epsilon_i$$

S6 Regression Tables

S6.1 Response Variable: Retweet count

Dep. Variable:	RetweetCount	No. Observations:	8256385
Model:	GLM	Df Residuals:	8256376
Model Family:	Poisson	Log-Likelihood:	-159360000
Deviance:	312020000	Pearson chi2:	85300000000

	coefficient	std. error	z
Intercept	0.732***	0.0	1567.78
Politics	0.252***	0.0	614.983
Health	0.171***	0.0	468.77
Personal	−0.221***	0.001	-302.112
Travel	−0.023***	0.001	-19.903
Economic	−0.288***	0.001	-553.027
CharacterCount	5.865***	0.003	2343.108
PunctuationCount	−20.636***	0.017	-1209.992
UpperCaseWordCount	1.823***	0.005	383.355

Table S11: Results of Regression 1 with the number of retweets as response. Exponentiated coefficients are plotted in Figure 2A. Note: *** p<0.01, ** p<0.05, * p<0.1

Dep. Variable:	RetweetCount	No. Observations:	8256385
Model:	GLM	Df Residuals:	8256364
Model Family:	Poisson	Log-Likelihood:	-135900000
Deviance:	265090000	Pearson chi2:	115000000000

	coefficient	std. error	z
Intercept	0.202***	0.001	343.199
Health Expert	0.104***	0.001	95.998
Influencer	2.483***	0.001	3689.186
Politics	0.039***	0.001	66.415
Politics x Health Expert	−0.035***	0.001	-27.059
Politics x Influencer	0.545***	0.001	685.773
Health	−0.048***	0.001	-91.49
Health x Health Expert	0.137***	0.001	121.982
Health x Influencer	0.068***	0.001	93.721
Personal	−0.138***	0.001	-150.581
Personal x Health Expert	0.399***	0.002	193.018
Personal x Influencer	0.253***	0.002	161.309
Travel	−0.326***	0.002	-178.026
Travel x Health Expert	0.581***	0.004	157.821
Travel x Influencer	−0.872***	0.003	-339.742
Economic	−0.338***	0.001	-456.267
Economic x Health Expert	0.016***	0.002	9.257
Economic x Influencer	−0.28***	0.001	-269.352
CharacterCount	6.952***	0.003	2637.001
PunctuationCount	−28.691***	0.019	-1531.376
UpperCaseWordCount	3.723***	0.005	821.569

Table S12: Results of Regression 2 with the number of retweets as response. Exponentiated coefficients are plotted in Figure 2B. Note: *** p<0.01, ** p<0.05, * p<0.1

Dep. Variable:	RetweetCount	No. Observations:	8256384
Model:	GLM	Df Residuals:	8256375
Model Family:	Poisson	Log-Likelihood:	-159330000
Deviance:	311950000	Pearson chi2:	83900000000

	coefficient	std. error	z
Novelty	1.319***	0.001	1528.269
CharacterCount	6.59***	0.002	2912.77
PunctuationCount	−21.866***	0.017	-1289.748
UpperCaseWordCount	1.871***	0.005	396.775
Politics x Novelty	0.575***	0.001	650.477
Health x Novelty	0.34***	0.001	442.156
Personal x Novelty	−0.44***	0.002	-273.775
Travel x Novelty	−0.071***	0.002	-28.711
Economic x Novelty	−0.623***	0.001	-553.214

Table S13: Results of Regression 3 with the number of retweets as response and 1-day Novelty as a predictor. Exponentiated coefficients are plotted in Figure 3A. Note: *** p<0.01, ** p<0.05, * p<0.1

Dep. Variable:	RetweetCount	No. Observations:	8256384
Model:	GLM	Df Residuals:	8256375
Model Family:	Poisson	Log-Likelihood:	-159740000
Deviance:	312780000	Pearson chi2:	86900000000

	coefficient	std. error	z
Novelty-3day	2.364***	0.002	1309.84
CharacterCount	7.525***	0.002	3606.544
PunctuationCount	−22.044***	0.016	-1337.167
UpperCaseWordCount	2.03***	0.005	440.557
Politics x Novelty-3day	1.286***	0.002	599.216
Health x Novelty-3day	0.6***	0.002	327.586
Personal x Novelty-3day	−1.148***	0.004	-280.221
Travel x Novelty-3day	−0.128***	0.006	-21.789
Economic x Novelty-3day	−1.591***	0.003	-567.584

Table S14: Results of Regression 3 with the number of retweets as response and 3-day Novelty as a predictor. Exponentiated coefficients are plotted in Figure 3A. Note: *** p<0.01, ** p<0.05, * p<0.1

Dep. Variable:	RetweetCount	No. Observations:	8256384
Model:	GLM	Df Residuals:	8256375
Model Family:	Poisson	Log-Likelihood:	-159690000
Deviance:	312690000	Pearson chi2:	86600000000

	coefficient	std. error	z
Novelty-7day	3.293***	0.003	1259.594
CharacterCount	7.862***	0.002	3921.979
PunctuationCount	−22.811***	0.016	-1389.518
UpperCaseWordCount	2.055***	0.005	449.205
Politics x Novelty-7day	2.358***	0.003	685.643
Health x Novelty-7day	1.05***	0.003	367.821
Personal x Novelty-7day	−1.555***	0.007	-226.889
Travel x Novelty-7day	−0.329***	0.009	-35.343
Economic x Novelty-7day	−2.454***	0.005	-530.072

Table S15: Results of Regression 3 with the number of retweets as response and 7-day Novelty as a predictor. Exponentiated coefficients are plotted in Figure 3A. Note: *** $p<0.01$, ** $p<0.05$, * $p<0.1$

Dep. Variable:	RetweetCount	No. Observations:	8091256
Model:	GLM	Df Residuals:	8091223
Model Family:	Poisson	Log-Likelihood:	-156650000
Deviance:	306690000	Pearson chi2:	76700000000

	coefficient	std. error	z
Novelty[Very Low]	0.537***	0.001	614.239
Novelty[Low]	0.626***	0.001	883.011
Novelty[Average]	0.819***	0.001	1135.881
Novelty[High]	0.683***	0.001	641.067
Novelty[Very High]	0.717***	0.002	400.753
CharacterCount	6.543***	0.003	1999.358
PunctuationCount	−22.052***	0.018	-1256.413
UpperCaseWordCount	1.918***	0.005	402.106
Politics x Novelty[Very Low]	0.232***	0.001	343.866
Politics x Novelty[Low]	0.25***	0.001	389.922
Politics x Novelty[Average]	0.299***	0.001	308.022
Politics x Novelty[High]	0.319***	0.002	171.222
Politics x Novelty[Very High]	0.535***	0.004	138.99
Health x Novelty[Very Low]	0.176***	0.001	270.23
Health x Novelty[Low]	0.232***	0.001	397.495
Health x Novelty[Average]	−0.008***	0.001	-9.492
Health x Novelty[High]	0.098***	0.001	73.066
Health x Novelty[Very High]	0.214***	0.002	85.858
Personal x Novelty[Very Low]	−0.45***	0.001	-368.387
Personal x Novelty[Low]	0.07***	0.001	67.159
Personal x Novelty[Average]	−0.583***	0.002	-237.731
Personal x Novelty[High]	0.106***	0.004	28.186
Personal x Novelty[Very High]	0.118***	0.008	15.389
Travel x Novelty[Very Low]	−0.078***	0.002	-37.843
Travel x Novelty[Low]	0.05***	0.002	32.892
Travel x Novelty[Average]	−0.194***	0.002	-82.501
Travel x Novelty[High]	−0.12***	0.004	-31.057
Travel x Novelty[Very High]	−0.131***	0.008	-16.913
Economic x Novelty[Very Low]	−0.227***	0.001	-259.185
Economic x Novelty[Low]	−0.267***	0.001	-332.227
Economic x Novelty[Average]	−0.417***	0.001	-326.319
Economic x Novelty[High]	−0.538***	0.003	-206.927
Economic x Novelty[Very High]	−0.52***	0.006	-84.518

Table S16: Results of Regression 3 with the number of retweets as response and Novelty levels as a predictor. Note: *** $p<0.01$, ** $p<0.05$, * $p<0.1$

Dep. Variable:	RetweetCount	No. Observations:	8256384
Model:	GLM	Df Residuals:	8256375
Model Family:	Poisson	Log-Likelihood:	-137360000
Deviance:	268010000	Pearson chi2:	114000000000

	coefficient	std. error	z
Health Expert	-0.912^{***}	0.005	-167.111
Influencer	3.392^{***}	0.003	1048.077
Novelty	2.649^{***}	0.006	469.323
Influencer x Novelty	-1.877^{***}	0.007	-273.336
Health Expert x Novelty	-0.196^{***}	0.011	-18.19
CharacterCount	7.906^{***}	0.003	2326.226
PunctuationCount	-31.456^{***}	0.019	-1697.47
UpperCaseWordCount	3.861^{***}	0.004	869.359

Table S17: Results of Regression 4 with the number of retweets as response and 1-day Novelty as a predictor. Exponentiated coefficients are plotted in Figure 3B. Note: *** p<0.01, ** p<0.05, * p<0.1

Dep. Variable:	RetweetCount	No. Observations:	8256384
Model:	GLM	Df Residuals:	8256375
Model Family:	Poisson	Log-Likelihood:	-137420000
Deviance:	268130000	Pearson chi2:	116000000000

	coefficient	std. error	z
Health Expert	0.205^{***}	0.002	108.64
Influencer	2.732^{***}	0.001	2464.621
Novelty-3day	1.31^{***}	0.004	307.256
Influencer x Novelty-3day	-1.204^{***}	0.006	-209.471
Health Expert x Novelty-3day	-0.594^{***}	0.009	-65.639
CharacterCount	7.3^{***}	0.003	2574.71
PunctuationCount	-30.645^{***}	0.018	-1658.4
UpperCaseWordCount	3.83^{***}	0.004	859.393

Table S18: Results of Regression 4 with the number of retweets as response and 3-day Novelty as a predictor. Exponentiated coefficients are plotted in Figure 3B. Note: *** p<0.01, ** p<0.05, * p<0.1

Dep. Variable:	RetweetCount	No. Observations:	8256384
Model:	GLM	Df Residuals:	8256375
Model Family:	Poisson	Log-Likelihood:	-137380000
Deviance:	268070000	Pearson chi2:	115000000000

	coefficient	std. error	z
Health Expert	0.024***	0.002	14.656
Influencer	2.711***	0.001	2930.336
Novelty-7day	2.327***	0.006	406.616
Influencer x Novelty-7day	−1.754***	0.008	-228.977
Health Expert x Novelty-7day	0.003	0.012	0.274
CharacterCount	7.603***	0.003	2475.415
PunctuationCount	−30.986***	0.018	-1678.8
UpperCaseWordCount	3.838***	0.004	863.215

Table S19: Results of Regression 4 with the number of retweets as response and 7-day Novelty as a predictor. Exponentiated coefficients are plotted in Figure 3B. Note: *** p<0.01, ** p<0.05, * p<0.1

Dep. Variable:	RetweetCount	No. Observations:	8091256
Model:	GLM	Df Residuals:	8091238
Model Family:	Poisson	Log-Likelihood:	-135150000
Deviance:	263700000	Pearson chi2:	110000000000

	coefficient	std. error	z
Intercept	−0.229***	0.001	-265.997
Health Expert	0.055***	0.001	47.258
Influencer	2.723***	0.001	4474.228
Novelty[Low]	0.22***	0.001	366.644
Novelty[Average]	0.38***	0.001	478.893
Novelty[High]	0.359***	0.001	322.309
Novelty[Very High]	0.593***	0.002	343.911
Influencer x Novelty[Low]	−0.295***	0.001	-366.122
Influencer x Novelty[Average]	−0.363***	0.001	-372.096
Influencer x Novelty[High]	−0.299***	0.001	-210.3
Influencer x Novelty[Very High]	−0.286***	0.003	-111.61
Health Expert x Novelty[Low]	0.016***	0.001	13.682
Health Expert x Novelty[Average]	−0.192***	0.002	-123.149
Health Expert x Novelty[High]	0.08***	0.002	36.39
Health Expert x Novelty[Very High]	0.115***	0.004	29.184
CharacterCount	7.98***	0.003	2372.22
PunctuationCount	−31.69***	0.019	-1694.245
UpperCaseWordCount	3.944***	0.004	880.757

Table S20: Results of Regression 4 with the number of retweets as response and Novelty levels as a predictor. Note: *** p<0.01, ** p<0.05, * p<0.1

S6.1.1 Response Variable: Is retweeted

Dep. Variable:	is_retweeted	No. Observations:	8256385
Model:	GLM	Df Residuals:	8256376
Model Family:	Poisson	Log-Likelihood:	-5117300
Deviance:	5690000	Pearson chi2:	5900000

	coefficient	std. error	z
Intercept	-1.845^{***}	0.002	-1090.212
Politics	-0.062^{***}	0.002	-34.312
Health	0.116^{***}	0.001	79.501
Personal	-0.295^{***}	0.003	-92.464
Travel	0.273^{***}	0.004	65.983
Economic	0.139^{***}	0.002	78.387
CharacterCount	1.921^{***}	0.009	218.79
PunctuationCount	7.314^{***}	0.034	215.585
UpperCaseWordCount	0.108^{***}	0.026	4.184

Table S21: Results of Regression 1 with the is_retweeted as response. Note: *** p<0.01, ** p<0.05, * p<0.1

Dep. Variable:	is_retweeted	No. Observations:	8256385
Model:	GLM	Df Residuals:	8256364
Model Family:	Poisson	Log-Likelihood:	-4932900
Deviance:	5321300	Pearson chi2:	5870000

	coefficient	std. error	z
Intercept	-2.003^{***}	0.002	-1083.062
Health Expert	0.277^{***}	0.004	65.628
Influencer	1.142^{***}	0.003	342.004
Politics	-0.053^{***}	0.002	-25.758
Politics x Health Expert	-0.055^{***}	0.006	-8.986
Politics x Influencer	0.089^{***}	0.005	19.71
Health	0.062^{***}	0.002	36.343
Health x Health Expert	-0.002	0.005	-0.498
Health x Influencer	0.006^{*}	0.004	1.649
Personal	-0.231^{***}	0.004	-66.012
Personal x Health Expert	0.025^{**}	0.01	2.338
Personal x Influencer	0.07^{***}	0.011	6.574
Travel	0.189^{***}	0.005	37.563
Travel x Health Expert	-0.152^{***}	0.015	-10.375
Travel x Influencer	-0.27^{***}	0.01	-27.713
Economic	0.136^{***}	0.002	65.843
Economic x Health Expert	-0.052^{***}	0.006	-8.838
Economic x Influencer	-0.176^{***}	0.004	-40.333
CharacterCount	2.001^{***}	0.009	221.823
PunctuationCount	6.99^{***}	0.036	192.192
UpperCaseWordCount	0.597^{***}	0.026	23.156

Table S22: Results of Regression 2 with the is_retweeted as response. Note: *** p<0.01, ** p<0.05, * p<0.1

Dep. Variable:	is_retweeted	No. Observations:	8256384
Model:	GLM	Df Residuals:	8256375
Model Family:	Poisson	Log-Likelihood:	-5117500
Deviance:	5690400	Pearson chi2:	5950000

	coefficient	std. error	z
Novelty	-3.386^{***}	0.003	-1008.034
CharacterCount	0.326^{***}	0.008	40.276
PunctuationCount	9.141^{***}	0.032	281.765
UpperCaseWordCount	-0.087^{***}	0.026	-3.329
Politics x Novelty	-0.128^{***}	0.004	-32.76
Health x Novelty	0.295^{***}	0.003	96.886
Personal x Novelty	-0.709^{***}	0.007	-100.579
Travel x Novelty	0.617^{***}	0.009	71.54
Economic x Novelty	0.281^{***}	0.004	74.301

Table S23: Results of Regression 3 with is_retweeted as response. Note: *** p<0.01, ** p<0.05, * p<0.1

Dep. Variable:	is_retweeted	No. Observations:	8091256
Model:	GLM	Df Residuals:	8091223
Model Family:	Poisson	Log-Likelihood:	-5025600
Deviance:	5572000	Pearson chi2:	5800000

	coefficient	std. error	z
Novelty[Very Low]	−1.633***	0.003	-474.027
Novelty[Low]	−1.669***	0.003	-623.529
Novelty[Average]	−1.839***	0.003	-660.415
Novelty[High]	−1.978***	0.004	-497.692
Novelty[Very High]	−2.122***	0.007	-293.921
CharacterCount	1.126***	0.013	86.004
PunctuationCount	10.136***	0.06	169.208
UpperCaseWordCount	−0.043	0.027	-1.631
Politics x Novelty[Very Low]	−0.15***	0.003	-48.0
Politics x Novelty[Low]	−0.057***	0.003	-20.307
Politics x Novelty[Average]	0.015***	0.004	3.505
Politics x Novelty[High]	0.066***	0.008	8.5
Politics x Novelty[Very High]	0.116***	0.019	6.199
Health x Novelty[Very Low]	0.026***	0.003	9.231
Health x Novelty[Low]	0.098***	0.002	42.277
Health x Novelty[Average]	0.18***	0.003	58.095
Health x Novelty[High]	0.232***	0.005	47.326
Health x Novelty[Very High]	0.333***	0.01	34.141
Personal x Novelty[Very Low]	−0.309***	0.005	-62.948
Personal x Novelty[Low]	−0.335***	0.005	-65.804
Personal x Novelty[Average]	−0.291***	0.009	-31.539
Personal x Novelty[High]	−0.25***	0.018	-13.661
Personal x Novelty[Very High]	−0.253***	0.041	-6.247
Travel x Novelty[Very Low]	0.053***	0.008	6.715
Travel x Novelty[Low]	0.095***	0.006	16.343
Travel x Novelty[Average]	0.197***	0.007	26.439
Travel x Novelty[High]	0.253***	0.012	21.259
Travel x Novelty[Very High]	0.38***	0.024	15.854
Economic x Novelty[Very Low]	0.135***	0.003	42.344
Economic x Novelty[Low]	0.137***	0.003	50.429
Economic x Novelty[Average]	0.08***	0.004	19.554
Economic x Novelty[High]	0.066***	0.007	8.871
Economic x Novelty[Very High]	0.093***	0.018	5.088

Table S24: Results of Regression 3 with is_retweeted as response and Novelty levels as a predictor. Note: *** p<0.01, ** p<0.05, * p<0.1

Dep. Variable:	is_retweeted	No. Observations:	8256384
Model:	GLM	Df Residuals:	8256375
Model Family:	Poisson	Log-Likelihood:	-4934600
Deviance:	5324500	Pearson chi2:	5890000

	coefficient	std. error	z
Intercept	−1.055***	0.011	-99.98
Health Expert	−0.469***	0.021	-22.581
Influencer	−0.331***	0.016	-20.699
Novelty	−1.725***	0.019	-88.604
Influencer x Novelty	3.039***	0.033	91.356
Health Expert x Novelty	−0.703***	0.042	-16.748
CharacterCount	1.348***	0.012	111.221
PunctuationCount	8.347***	0.035	238.593
UpperCaseWordCount	0.417***	0.026	16.084

Table S25: Results of Regression 4 with is_retweeted as response. Note: *** p<0.01, ** p<0.05, * p<0.1

Dep. Variable:	is_retweeted	No. Observations:	8091256
Model:	GLM	Df Residuals:	8091238
Model Family:	Poisson	Log-Likelihood:	-4850100
Deviance:	5221100	Pearson chi2:	5780000

	coefficient	std. error	z
Intercept	−1.828***	0.003	-606.629
Health Expert	−1.557***	0.005	-343.154
Influencer	0.982***	0.003	284.819
Novelty[Low]	0.008***	0.002	4.011
Novelty[Average]	−0.148***	0.003	-55.305
Novelty[High]	−0.267***	0.004	-73.622
Novelty[Very High]	−0.338***	0.006	-55.805
Influencer x Novelty[Low]	0.035***	0.004	7.985
Influencer x Novelty[Average]	0.26***	0.005	53.995
Influencer x Novelty[High]	0.451***	0.006	70.64
Influencer x Novelty[Very High]	0.532***	0.012	43.443
Health Expert x Novelty[Low]	−0.011**	0.005	-2.19
Health Expert x Novelty[Average]	−0.032***	0.006	-5.202
Health Expert x Novelty[High]	−0.064***	0.009	-7.358
Health Expert x Novelty[Very High]	−0.018	0.017	-1.038
CharacterCount	1.186***	0.013	91.642
PunctuationCount	10.631***	0.06	177.689
UpperCaseWordCount	0.32***	0.027	12.013

Table S26: Results of Regression 4 with is_retweeted as response and Novelty levels as a predictor. Note: *** p<0.01, ** p<0.05, * p<0.1

S6.1.2 Quadratic relationship between Novelty and Retweet count

Dep. Variable:	RetweetCount	No. Observations:	8256384
Model:	GLM	Df Residuals:	8256382
Model Family:	Poisson	Log-Likelihood:	-162260000
Deviance:	317820000	Pearson chi2:	90200000000

	coefficient	std. error	z
Intercept	1.344^{***}	0.0	6862.278
Novelty	-2.48^{***}	0.002	-1533.193

Table S27: Results of Regression with RetweetCount as a response and Novelty as a predictor. Note: *** p<0.01, ** p<0.05, * p<0.1

Dep. Variable:	RetweetCount	No. Observations:	8256384
Model:	GLM	Df Residuals:	8256382
Model Family:	Poisson	Log-Likelihood:	-163490000
Deviance:	320270000	Pearson chi2:	86100000000

	coefficient	std. error	z
Intercept	1.462^{***}	0.0	7259.216
Novelty^2	0.241^{***}	0.008	29.52

Table S28: Results of Regression with RetweetCount as a response and square of Novelty as a predictor. Note: *** p<0.01, ** p<0.05, * p<0.1

Dep. Variable:	RetweetCount	No. Observations:	8256384
Model:	GLM	Df Residuals:	8256381
Model Family:	Poisson	Log-Likelihood:	-162230000
Deviance:	317760000	Pearson chi2:	90300000000

	coefficient	std. error	z
Intercept	1.363^{***}	0.0	6294.467
Novelty	-2.646^{***}	0.002	-1462.142
Novelty^2	-2.119^{***}	0.009	-228.304

Table S29: Results of Regression with RetweetCount as a response and, Novelty and square of Novelty as predictors. Note: *** p<0.01, ** p<0.05, * p<0.1

S7 Sample of highly reshared tweets

In the tables bellow we present a sample of tweets with very high number of retweets. In Figure 2B, we find that personal, and health-related tweets posted by health experts and, political tweets posted by influencers are retweeted more than other tweets. Additionally, in Figure 3A we observe that novel information is being spread more than non-novel information for all the social dimensions. Moving beyond the numbers, we provide the following sample of tweets that fall into the aforementioned groups.

Tweet
COVID 19 is the worst disease process I've ever worked with in my 8 years as an ICU nurse.
I can't believe people are still arguing about wearing masks. My mother died of COVID last Sunday. My father is going to follow this weekend. I'm not asking for your condolences. I'm asking you to wear a fucking mask.
I don't usually rant on social media. However, after a week of service in the ICU, I'd like to get a few things off my chest. #COVID19 (1/10)
You might hear people saying it isn't real. It is. You might hear people saying it isn't bad. It is. You might hear people saying it can't take you down. It can. I survived Ebola. I fear #COVID-19. Do your part. Stay home. Stay safe. And every day I'll come to work for you

Table S30: Sample of highly retweeted personal-related tweets posted by health experts

Tweet
Update. The Cleveland Clinic has developed a COVID 19 test that gives results in 8 hours as opposed to the 2-3 days it takes for the other tests. They developed the test in NINE days, I repeat nine. Scientists worked 24/7 once the CDC gave them the okay on 3/2.
Please circulate this: The French government has issued a warning against taking anti-inflammatory drugs (ibuprofen, Advil, cortisone) with covid-19 symptoms, as it may cause severe cases of the disease, even in young and middle aged adults with no underlying conditions
Korea finished developing the 10 minute Covid-19 diagnostic kit and is now ramping up production. They plan to export 300.000 test-kits per week - https://t.co/DpJCph9RT7

Table S31: Sample of highly retweeted health-related tweets posted by health experts

Tweet
I don't think people are digesting the fact that our President did NOTHING to stop coronavirus and is now saying 200,000 deaths would be a success.
It arrived in South Korea ON THE SAME FUCKING DAY as the US, they took it very seriously and in total only *158* people have died.
BREAKING: Many leading Chinese scientists are starting to speak out and say COVID-19 (coronavirus) originated at a government bioweapons research facility in Wuhan, rather than the widely-held belief that it emerged from the city's Huanan seafood markets. https://t.co/DsxwJSAox2 https://t.co/TbshN8eDni
The Democrat Governor of Nevada hates Trump so much that he's banning Wuhan Virus patients from a potential life-saving treatment. This is a new low in a week of new lows. These people are filth. ¡emoji¿ https://t.co/Y2OYXnE72p

Table S32: Sample of highly retweeted politics-related tweets posted by influencers

Tweet
Race is on as Australian researchers rush to make coronavirus vaccine https://t.co/iCnIbYkUpb
Whole genome of the Wuhan coronavirus, 2019-nCoV, sequenced https://t.co/UdH0s0KvXe
Only 10% of Chinese cases and 25% of international cases currently being detected. #2019nCoV #coronavirus https://t.co/zlPUSpoTt8)
The world has ongoing outbreaks of #Measles #Ebola #Cholera #influenza not only #coronavirus #nCoV

Table S33: Sample of highly retweeted tweets with high novelty of information

References

[1] Aral, S. and Dhillon, P. (2016). Unpacking novelty: The anatomy of vision advantages. *Available at SSRN 2388254*.

[2] Blei, D. M., Ng, A. Y., and Jordan, M. I. (2003). Latent dirichlet allocation. *the Journal of machine Learning research*, **3**, 993–1022.

[3] Chinazzi, M., Davis, J. T., Ajelli, M., Gioannini, C., Litvinova, M., Merler, S., y Piontti, A. P., Mu, K., Rossi, L., Sun, K., *et al.* (2020). The effect of travel restrictions on the spread of the 2019 novel coronavirus (covid-19) outbreak. *Science*, **368**(6489), 395–400.

[4] Compton, R., Jurgens, D., and Allen, D. (2014). Geotagging one hundred million twitter accounts with total variation minimization. In *2014 IEEE international conference on Big data (big data)*, pages 393–401. IEEE.

[5] Gallotti, R., Valle, F., Castaldo, N., Sacco, P., and De Domenico, M. (2020).

Assessing the risks of 'infodemics' in response to covid-19 epidemics. *Nature Human Behaviour*, **4**(12), 1285–1293.

[6] Le, Q. and Mikolov, T. (2014). Distributed representations of sentences and documents. In *International conference on machine learning*, pages 1188–1196. PMLR.

[7] Mikolov, T., Sutskever, I., Chen, K., Corrado, G., and Dean, J. (2013). Distributed representations of words and phrases and their compositionality. *arXiv preprint arXiv:1310.4546*.

[8] Singh, L., Bansal, S., Bode, L., Budak, C., Chi, G., Kawintiranon, K., Padden, C., Vanarsdall, R., Vraga, E., and Wang, Y. (2020). A first look at covid-19 information and misinformation sharing on twitter. *arXiv preprint arXiv:2003.13907*.

[9] Vosoughi, S., Roy, D., and Aral, S. (2018). The spread of true and false news online. *Science*, **359**(6380), 1146–1151.

[10] Wang, Z., Hale, S., Adelani, D. I., Grabowicz, P., Hartman, T., Flöck, F., and Jurgens, D. (2019). Demographic inference and representative population estimates from multilingual social media data. WWW '19, page 2056–2067, New York, NY, USA. Association for Computing Machinery.

[11] Zafar, M. B., Bhattacharya, P., Ganguly, N., Gummadi, K. P., and Ghosh, S. (2015). Sampling content from online social networks: Comparing random vs. expert sampling of the twitter stream. *ACM Transactions on the Web (TWEB)*, **9**(3), 1–33.
